# Scale-up of a comprehensive model to improve tuberculosis control in China: lessons learned and the way forward

**DOI:** 10.1186/s40249-021-00828-1

**Published:** 2021-03-25

**Authors:** Qian Long, Fei Huang, Shi-Tong Huan, Yan-Lin Zhao

**Affiliations:** 1 Global Health Research Center, Duke Kunshan University, Jiangsu, China; 2 National Center for Tuberculosis Control and Prevention, China CDC, No.155 Changbai Road, Changping District, Beijing, China; 3 Bill & Melinda Gates Foundation, China Office, Beijing, China

## Background

The World Health Organization’s End TB Strategy (2016–2035) sets the goal of ending the tuberculosis (TB) epidemic, in line with the United Nations Sustainable Development Goals [1, 2]. In addition to setting indicators for measuring the decline in TB incidence and mortality, the End TB Strategy highlights the need to alleviate the financial hardship faced by many TB-affected families [1]. The international community has acknowledged that progress toward universal health coverage with a “whole system” approach will be fundamental to achieving the ambitious goal of ending TB globally. Many low- and middle-income countries with high TB burden struggle to afford and acquire new tools (e.g., new technologies and drugs for diagnosis and treatment) and adapt their health systems to strengthen TB control.

China has the third-highest burden of TB and second-highest burden of multidrug-resistant TB (MDR-TB) globally. Yet between 1990 and 2010, the prevalence rate of smear-positive pulmonary TB in China declined significantly [3]. This achievement was largely attributable to nationwide coverage of a World Health Organization–recommended directly observed treatment, short-course (DOTS) strategy that was implemented through a vertical TB control program led by the Chinese Center for Disease Control and Prevention (China CDC) [3]. However, the challenges of TB control in China continue to include an increase in MDR-TB patients; poor patient adherence to TB treatment, particularly MDR-TB treatment; patients’ difficulty in affording TB and MDR-TB care; and low efficiency of TB service delivery [3].

**Fig. 1:**
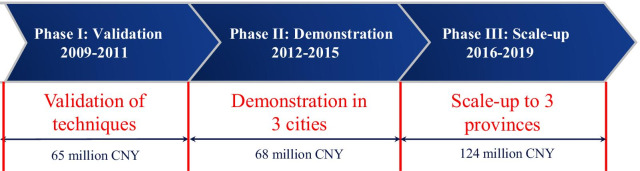
China National Health Commission and Gates Foundation TB Prevention Control Project phases, 2009–2019

The National Health Commission (NHC) of China, formerly the Ministry of Health, oversees the national program for TB and MDR-TB prevention and control in China. With support from the Bill & Melinda Gates Foundation, the NHC has, since 2009, developed an innovative program to strengthen TB control, and the China CDC led the implementation. This program, the “China National Health Commission and Gates Foundation TB Prevention and Control Project” (China-Gates TB project), was carried out in three phases, from 2009 to 2019 (Fig. 1).

In the context of deepening China’s health system reform, the China-Gates TB project introduced new policies, tools, and service delivery approaches to improve the diagnosis and treatment of TB and MDR-TB. Phase I, which was carried out in four cities between 2009 and 2011, centered on the introduction of new tools to improve TB (particularly MDR-TB) diagnosis, treatment, and patient management. The tools included a variety of new diagnostics (e.g., molecular tests) that were more sensitive, and prompt for diagnosis, made standardization of MDR-TB diagnostic and treatment packages more feasible, and opened the doors for use of electronic medication monitors (EMMs) to improve treatment adherence of TB patients.

In February 2013, the NHC issued Decree No. 92, which describes the process of integrating TB clinical services and patient management into China’s tiered health service delivery system. At the same time, basic medical insurance schemes were expected to be a primary source to pay for TB care in the hospitals. With the transition to a more integrated and collaborative TB control model in China, Phase II of the project (2012–2015), which was implemented in three prefectures in eastern, central, and western provinces of China, demonstrated how to ensure that innovative diagnostic, treatment, and financing approaches for all TB patients could be effectively adapted to the new model. Key findings from Phases I and II were published in several peer-reviewed international journals [4–7].

Based on the achievements of Phases I and II, Phase III was kicked off in 2016 in the provinces of Zhejiang, Ningxia, and Jilin. This phase aimed to expand the comprehensive model of TB control at the provincial level and introduce new policies, technologies, and approaches to cope with the challenges identified in the previous phases and thus improve the performance of TB control in China. Phase III centered on three domains. The first domain focused on strengthening the TB care delivery system, including (a) planning, policy development, and advocacy to support scale-up of the comprehensive TB control model through local government commitment; (b) capacity-building of TB care professionals through innovative training approaches; and (c) developing a new TB surveillance and information system. The second domain focused on improving the quality of TB care, including the (a) upgrade of laboratory capacity for TB and MDR-TB diagnostics; (b) standardization of diagnosis, treatment, and patient management of TB and MDR-TB; (c) scale-up of using EMMs to improve community-based case management; and (d) introduction of new anti-TB drugs. The third domain focused on improving the mechanism for financing TB care and included (a) introducing multisource financing for TB clinical care, (b) exploring the reform of payment methods for TB clinical care, and (c) simplifying the process of medical assistance for patients who are eligible to benefit.

## Overview of the findings

Duke Global Health Institute and Duke Kunshan University (a Chinese-American partnership of Wuhan University and Duke University), as a third party, led the monitoring, learning, and evaluation of Phase III of the China-Gates project. The evaluation team consisted of collaborators from Peking University, Fudan University, Zhejiang University, and China CDC. The evaluation team undertook performance-based monitoring, using routine data from all counties of the three project provinces and an in-depth site evaluation in the selected prefectures in each province. For each province, two prefectures were selected, representing a variety of socioeconomic levels. Within each prefecture, two counties were selected using the same criteria; in total, six prefectures and twelve counties were involved in the site evaluation. The team applied both quantitative and qualitative methods for the baseline and final evaluation. Quantitative data collection included a patient survey with TB and MDR-TB patients, a survey with TB professionals, an institution-based survey for TB-designated hospitals and local CDCs, as well as routine data from the health information system in the TB-designated hospitals, medical records of TB and MDR-TB patients, and surveillance data on TB and MDR-TB diagnosis, treatment, and case management. Qualitative data included data extracted from local policy documents; qualitative interviews with decision-makers and key informants from the local health authority, health insurance agency, CDC, TB-designated hospitals, and focus group discussions with TB care providers and TB and MDR-TB patients; site observations; and field notes.

### Quality of TB and MDR-TB care

The quality of diagnosis and treatment of TB and MDR-TB significantly improved in the project provinces, which is, to a great extent, attributable to the availability and accessibility of new diagnostic technologies and standardization of treatment procedures. There was a notable increase of bacteriologically confirmed pulmonary TB cases and a rise in the proportion of TB and DR-TB patients receiving the recommended diagnostic services and clinical treatment at the time of project final evaluation in 2019 compared to the baseline study in 2016. However, irrational use of second-line anti-TB drugs for TB treatment remained, and varied in magnitude, across the study sites. The qualitative results reveal that the achievements were mainly attributed to successful scale-up of the new rapid diagnostic technologies as well as training and monitoring of TB clinical services. Meanwhile, the qualitative findings raise concerns on the sustainability and affordability of applying new diagnostic technologies. In terms of appropriate application of new anti-TB drugs, the safety profile of bedaquiline-containing treatment for patients with MDR-TB and extensively drug-resistant TB (XDR-TB) was examined [8]. The result shows that bedaquiline was generally well-tolerated by MDR-TB patients, and support the use of bedaquiline for treatment of MDR-TB and XDR-TB. In addition, the TB treatment success rate slightly increased with the expansion of coverage of EMMs in the project provinces. The further study is proposed to test improved EMMs with real-time feedback, according to the knowledge gained from the implementation [9].

### Financing for TB care

A mechanism of multisource financing for TB and MDR-TB treatment has been introduced in the project provinces. However, the introduction was initially delayed, and thus many households with a DR-TB patient suffered from a catastrophic health expenditure at the time of project final evaluation. Strengthening cooperation among multiple sectors and improving the accountability of different government agencies will be the key to ensure significant progress toward alleviating the financial burden of TB and MDR-TB patients [10]. In addition, paying for TB treatment based on a capitation method has been piloted. The result shows a decrease in the total medical expenditure of a full treatment course of TB and a savings for the health insurance fund on TB treatment.

### TB care delivery system

Capacity-building of TB professionals through e-learning and reform of the TB information system are two approaches to strengthening the TB care delivery system. The scale-up of e-learning for continuing TB medical education shows that TB clinical doctors and primary health care providers benefited from e-learning and applied what they learned in routine practice, while, the training for public health physicians needed improvement. Challenges in promoting of e-learning in continuing medical education included unmet learning needs, weak leadership, unfriendly environment, and lack of incentives to TB professionals participating in the e-learning [11]. In addition, the pilot of a new TB surveillance system in the project provinces demonstrated how the diverse infrastructures of the information system could be reformed to achieve the functions of automatic data extraction and data exchange and better cater to the needs of healthcare professionals [12].

## Lessons learned and the way forward

With the support of local government in the project provinces, the scale-up of new diagnostics and training and monitoring of TB clinical services contributed to improvement in the quality of diagnosis and treatment of TB and MDR-TB. Yet coordination of multisource financing for TB care is the most challenging component due to the various interests of stakeholders, weak leadership, and poor engagement of multiple sectors. Knowledge gained from introduction of e-learning for TB training and a pilot of reform of the TB information system both indicated the need for investment in infrastructure and human resources.

Furthermore, the COVID-19 pandemic has had a devastating effect on TB responses globally. Health system overload from treating COVID-19 cases and preventing transmission has, to some extent, resulted in a decrease of, or limit on, anti-TB services delivery. Similarly, the economic recession caused by the pandemic has had a negative impact on the generation of health resources. Given the potential impact of COVID-19 on TB control in the near future, improving performance of TB control should be in line with government efforts toward the development of effective universal health coverage in China.

The authors hope that the experience and findings from the China-Gates TB project can contribute to successful achievement of innovative approaches for TB control in other low- and middle-income countries with a high TB burden.

## Data Availability

Not applicable.

## Abbreviations

CDC: Center for Disease Control and Prevention
DOTS: Directly observed treatment, short-course
DR-TB: Drug-resistant tuberculosis
EMM: Electronic medication monitor
MDR-TB: Multidrug-resistant tuberculosis
NHC: National Health Commission
TB: Tuberculosis
WHO: World Health Organization
XDR-TB: Extensively drug-resistant tuberculosis
